# IL-23 receptor deficiency results in lower bone mass via indirect regulation of bone formation

**DOI:** 10.1038/s41598-021-89625-2

**Published:** 2021-05-13

**Authors:** Wida Razawy, Celso H. Alves, Marijke Koedam, Patrick S. Asmawidjaja, Adriana M. C. Mus, Mohamed Oukka, Pieter J. M. Leenen, Jenny A. Visser, Bram C. J. van der Eerden, Erik Lubberts

**Affiliations:** 1 Department of Rheumatology, Erasmus MC, University Medical Center Rotterdam, Dr. Molewaterplein 40, 3015 GD Rotterdam, The Netherlands; 2 Department of Immunology, Erasmus MC, University Medical Center Rotterdam, Rotterdam, The Netherlands; 3 Department of Internal Medicine, Erasmus MC, University Medical Center Rotterdam, Rotterdam, The Netherlands; 4 Department of Pediatrics, Seattle Children’s Research Institute, Center for Immunity and Immunotherapies, Seattle, USA; 5 Department of Immunology, University of Washington, Seattle, USA; 6 Present Address: Center for Innovative Biomedicine and Biotechnology (CIBB), University of Coimbra, Coimbra, Portugal; 7 Present Address: Coimbra Institute for Clinical and Biomedical Research (iCBR), Faculty of Medicine, University of Coimbra, Coimbra, Portugal; 8 Present Address: Association for Innovation and Biomedical Research On Light and Image (AIBILI), Coimbra, Portugal; 9 Present Address: Clinical Academic Center of Coimbra (CACC), Coimbra, Portugal

**Keywords:** Immunology, Medical research, Rheumatology

## Abstract

The IL-23 receptor (IL-23R) signaling pathway has pleiotropic effects on the differentiation of osteoclasts and osteoblasts, since it can inhibit or stimulate these processes via different pathways. However, the potential role of this pathway in the regulation of bone homeostasis remains elusive. Therefore, we studied the role of IL-23R signaling in physiological bone remodeling using IL-23R deficient mice. Using µCT, we demonstrate that 7-week-old IL-23R^−/−^ mice have similar bone mass as age matched littermate control mice. In contrast, 12-week-old IL-23R^−/−^ mice have significantly lower trabecular and cortical bone mass, shorter femurs and more fragile bones. At the age of 26 weeks, there were no differences in trabecular bone mass and femur length, but most of cortical bone mass parameters remain significantly lower in IL-23R^−/−^ mice. In vitro osteoclast differentiation and resorption capacity of 7- and 12-week-old IL-23R^−/−^ mice are similar to WT. However, serum levels of the bone formation marker, PINP, are significantly lower in 12-week-old IL-23R^−/−^ mice, but similar to WT at 7 and 26 weeks. Interestingly, *Il23r* gene expression was not detected in in vitro cultured osteoblasts, suggesting an indirect effect of IL-23R. In conclusion, IL-23R deficiency results in temporal and long-term changes in bone growth via regulation of bone formation.

## Introduction

Bone homeostasis is maintained by bone resorbing osteoclasts and bone forming osteoblasts^1^. The importance of maintenance of bone homeostasis is underlined by diseases where imbalance in bone formation and degradation occurs. In many cases, disruption of bone homeostasis may be caused due to an imbalanced immune system^2^. Examples of inflammatory diseases accompanied with extensive systemic and local bone degradation include rheumatoid arthritis^3,4^ and psoriatic arthritis (PsA)^5^. In contrast, ankylosing spondylitis is associated with early process of systemic bone loss, but demonstrates extensive bone formation in the spine and periphery at later stages^6^.

Notably, in both extremes, a role for Interleukin-23 (IL-23) has been reported^6–8^. IL-23 belongs to the IL-12 cytokine family, and is composed of a heterodimer of the subunits IL-23p19 and IL-12p40^9,10^. The receptor for IL-23 (IL-23R) is formed by the subunits IL-23R and IL-12Rβ1^11,12^. Due to its role in induction of other pro-inflammatory cytokines, such as IL-17A, GM-CSF, IL-22, the IL-23R signaling pathway has been the subject of interest in different immune-mediated inflammatory diseases accompanied with bone erosions^13^.

In this context, patients with rheumatoid arthritis and psoriatic arthritis have increased levels of IL-23 in their serum^7,8^. In mice, systemic overexpression of IL-23 via hydrodynamic delivery of IL-23 minicircle DNA, induces chronic arthritis, increases osteoclast differentiation and systemic bone loss^14^. Similarly, a psoriasis-like disease develops in the novel K23 mouse model, which suffer from increased levels of IL-23 in the skin^15^. In these mice, the psoriasis-like disease proceeds psoriatic arthritis including enthesitis, dactylitis and bone destruction. Interestingly, overexpression of IL-23 via IL-23 minicircle DNA in a murine model of spondyloarthropathy, leads to pathological new bone formation during the initial phase of disease, while destruction of articular surfaces occurs at later time points^16^. These studies indicate that increased levels of IL-23 result to inflammatory conditions accompanied with excessive bone formation and/or degradation.

On the other hand, studies have demonstrated that absence of IL-23 also affects bone physiology. Reduced trabecular bone mineral density was detected in 12- and 26-week-old IL-23p19^−/−^ mice^17^. Other studies did not find any bone abnormalities in 8–^14^ and 12-week-old^18^ IL-23p19^−/−^ mice, but reported higher trabecular number (Tb.N) in 26-week-old IL-23p19^−/−^ mice^14^. Yet a recent study demonstrated higher trabecular bone mass in 2- and 12-months-old IL-12p40^−/−^ mice, which lack both IL-12 and IL-23^19^. Clearly, IL-23 deficiency results in altered bone physiology, however the lack of consensus in the findings emphasizes the need for additional in vivo studies to unravel the precise role of IL-23 deficiency herein.

In this attempt, knowledge gained from in vitro studies, which have demonstrated both direct or indirect effects of IL-23 on osteoblasts and osteoclasts is valuable^20^. While one study demonstrated that IL-23 can promote osteoclast formation by upregulation of RANK on bone marrow (BM)-derived osteoclast precursors^21^, another study demonstrated inhibitory effects of IL-23 on osteoclast formation through induction of GM-CSF in T cells^17^. It should be noted that although most studies use the whole BM population for differentiation of osteoclasts, it was demonstrated earlier that only the early blasts (CD31^+^Ly6C^−^), myeloid blasts (CD31^+^Ly6C^+^) and monocytes (CD31^−^Ly6c^+^) differentiated towards osteoclasts^22^. From these three, myeloid blasts appeared to be the most potent in osteoclastogenesis.

In primary osteoblasts, IL-23R protein is absent, and IL-23 treatment does not influence alkaline phosphatase (ALP) activity, RANKL expression and the proliferation of these cells^23^. However, signals of the IL-23R pathway can affect osteoblasts indirectly through IL-17A and IL-22, since their receptors are present on osteoblasts. Indeed, IL-17A inhibits ALP activity of osteoblasts^24^, while IL-22 stimulates mesenchymal stem cell migration and osteogenesis-related genes^25^. The above studies demonstrate the complexity of the interplay between IL-23 and cells of the bone. Adding to this complexity, bone physiology is influenced by different factors such as endocrine hormones^26^ or fat metabolism proteins such as leptin^27^, which could also affect IL-23 levels^28,29^.

Despite the ample amount of data suggesting pleiotropic effects of the IL-23R pathway and its downstream cytokines on bone cells, the effects of IL-23R signaling on bone remodeling during steady state are not well defined. We studied the role of IL-23R signaling in bone homeostasis at different ages using IL-23R deficient mice, and demonstrate that IL-23R deficiency results in changes in bone mass via indirect regulation of osteoblast function.

## Material and methods

### Animals

Knock-in IL-23R-GFP reporter (IL-23R^GFP/+^) mice were kindly provided by Dr. Mohamed Oukka, Seattle, USA and Prof. Dr. Vijay Kuchroo, Boston, USA^30^. For generation of IL-23R^GFP/+^ mice, an IRES-GFP cassette was introduced after exon 8 of the endogenous IL-23R gene. The targeting construct was electroporated into Bruce4 ES cells. Targeted ES cells were injected into BALB/c blastocysts and male chimeras were bred with female C57BL/6 mice^30^. IL-23R^GFP/+^ mice were bred to generate IL-23R^−/−^ (IL-23R^GFP/GFP^) and WT (IL-23R^+/+^) mice in the Erasmus MC experimental animal facility. Seven-, 12- and 26-week-old littermate male mice were used for this study. All mice were kept under specific pathogen-free conditions at the Erasmus MC experimental animal facility. Food and water were provided ad libitum. All animal experiments were performed in accordance with relevant guidelines and regulations and were approved by the Erasmus MC Dutch Animal Ethics Committee (DEC).

### Micro-computed tomography (μCT)

The left femurs were dissected and fixed overnight in 10% formalin at 4 ° C. The bones were then stored in 70% ethanol at 4 °C until microcomputed tomography (μCT) analysis was performed using a SkyScan 1076 at a 9 μm voxel resolution and 2300 ms exposure time. The following settings were used: X-ray power of 40 kV and tube current of 250 mA. Beam hardening (20%) was reduced using a 1 mm aluminum filter, ring-artefacts was set at 5 and an average of three photos (frame averaging) at each angle (0.8°) was taken to generate the final images. For the analysis of trabecular bone parameters, the distal metaphysis was scanned (a scan area of 1.35 mm from the distal growth plate towards femoral center). Analysis of the cortical bone parameters was performed in the diaphyseal cortex, which comprised a scan area of 0.45 mm in the femoral center. 3D reconstruction and data analysis were performed using manufacturer-provided software from Bruker MicroCT (NRecon, Data viewer, CT analyzer, SkyScan). Analyzed trabecular and cortical bone parameters are depicted according to the ‘Guidelines for assessment of bone microstructure in rodents using micro-computed tomography’ of the American society for bone and mineral research^31^.

### Three-point bending test

The same femurs used for the μCT analysis were subjected to a three-point bending test using a Chatillon TCD225 series force measurement system (Technex BV, The Netherlands)^32^. Displacement (mm) and force (N) were registered. Stiffness (N/mm) and work-to-failure (total amount of energy required to fracture, indicated by area under the curve for load and distance, Nmm) were calculated.

### Flow cytometry

Monoclonal antibody stainings of BM cells were performed as described previously^33^. Briefly, BM cells were incubated for 30 min with 50 μl anti-FCγRII/III antibodies (Bioceros) to block non-specific binding. Cells were subsequently incubated for 30 min with anti-mouse CD31 (Biorad) and Ly6C (BioLegend) antibodies. For exclusion of dead cells, BM cells were incubated with Fixable Viability Dye eFluor506 (eBioscience) for 30 min. All incubation steps were performed at 4 °C in the dark. Samples were acquired on an LSRII flow cytometer (BD Biosciences), and data were analyzed using FlowJo v7.6 software (Tree Star Inc. Ashland, OR).

### Cell culture

To obtain osteoclasts, BM cells from femurs and tibia were cultured for 5–9 days in the presence of 30 ng/ml recombinant M-CSF (R&D Systems) and 20 ng/ml recombinant RANKL-TEC (R&D Systems). Cells were seeded in 96-well flat-bottom plates at a density of 1.0 × 10^5^ BM cells/well in α-MEM (ThermoFisher Scientific) supplemented with 10% fetal calf serum, 100 U/ml penicillin/ streptomycin (Lonza) and 250 ng/ml amphotericine B/fungizone (Antibiotic antimycotic solution, Sigma). The medium was refreshed every 3 days. For the bone resorption assays, cells were cultured in a Corning osteoassay surface plate (Corning, USA).

To differentiate osteoblasts, 1.0 × 10^6^ BM cells were cultured in α-MEM supplemented with 15% fetal calf serum, 100 U/ml penicillin/streptomycin and 250 ng/ml amphotericine B/fungizone in 24-well plates. Half of the medium was refreshed every 3 or 4 days and l-ascorbic acid (Sigma-Aldrich) and β-glycerophosphate (Sigma-Aldrich) were added to the medium. At day three, 0.1 mM of l-ascorbic acid and 0.01 M of β-glycerophosphate were added. During subsequent medium refreshments, 0.05 mM of l-ascorbic acid and 5 mM of β-glycerophosphate were added.

### Tartrate-resistant acid phosphatase (TRAP) assay

Cells were washed with PBS and fixed with 10% formalin. TRAP^+^ cells were stained using a TRAP leukocyte kit (Sigma-Aldrich). The staining was performed according to manufacturer’s instructions, except the following adaptation: to visualize osteoclasts specifically, we used 1 M tartrate solution instead of the 0.3 M recommended by the manufacturer. Per well, 7 photos were taken at different locations to minimize the effect of unequal osteoclast development across the wells. Osteoclasts with ≥ 3 nuclei were counted using the online available ImageJ software (https://imagej.net/Welcome).

### Bone resorption assay

The supernatant of the cells was removed, and the wells were washed three times with distilled water to lyse cells. The wells were stained with 5% silver nitrate (Sigma-Aldrich) in bright daylight for 30 min. The wells were subsequently fixed for 40–60 s in 5% sodium carbonate (Merck) solubilized in 25% formalin. Lastly, the wells were incubated for 2 min in 5% sodium thiosulphate (Merck) in deionized water. After each incubation step, wells were washed three times with distilled water. Per well, 4 photos were taken to minimize the effect of unequal resorption activity across the wells. Bone resorption was quantified by measuring the percentage resorbed area per photo, using the online available ImageJ software (https://imagej.net/Welcome). The mean percentage of the 4 photos was used to determine osteoclast activity in each well.

### Real-time PCR

RNA was isolated from osteoblasts at day 10 of culture, using the GenElute Mammalian Total RNA Miniprep Kit according to the manufacturer’s instructions (Sigma Aldrich). RNA was treated with 0.1 U/μl DNAse I Amplification Grade (Invitrogen). cDNA was synthesized using random hexamer primers, oligo(dT) primers and 10 U/μl Superscript II (Invitrogen). Primer sequences and probes are listed in Table 1. Real-time PCR was performed using the Viia7 (Applied Biosystems) system.

**Table 1:** Primer sequences and probes used for RT-PCRs.

Gene	Primer 1	Primer 2	Probe #
*Gapdh*	AGCTTGTCATCAACGGGAAG	TTTGATGTTAGTGGGGTCTCG	9
*Il23r*	CCAAGTATATTGTGCATGTGAAGA	AGCTTGAGGCAAGATATTGTTGT	94
*Il12rβ1*	GCTTGGGAACCGAACCAT	GGAGGGGTCGTCTTGGTC	95

### ELISA

Leptin and testosterone were measured in serum samples using the Leptin or the Testosterone mouse/rat ELISA (Alpco Diagnostics). Procollagen I N-terminal propeptide (PINP) was measured using the mouse PINP ELISA (Abbexa), and Anti-Müllerian Hormone (AMH) was measured using the mouse AMH ELISA (Ansh Labs). ELISA’s were performed according to manufacturer’s instructions.

## Statistical analysis

Data are expressed as mean ± SEM. Data were tested for normality with Kolmogorov–Smirnov method using IBM SPSS Statistics 24. Statistical difference between groups was assessed using unpaired t tests.

To assess interactions between age (7, 12, 26 weeks) and genotype (WT and IL-23R^−/−^) of mice, two-way ANOVA was performed. In case of significant interaction between age and genotype, unpaired t test was performed to compare WT vs IL-23R^−/−^ at each age. One-way ANOVA with Tukey’s multiple comparison test was used to assess differences within each genotype over time (7, 12 and 26 weeks). In case of no significant interaction between age and genotype according to two-way ANOVA, but a significant difference for only one of the parameters age or genotype, the steps were performed as explained above only for the parameter that came out as significantly different. Statistical differences were determined using GraphPad Prism version 5.01 (GraphPad Software) and p values < 0.05 were considered statistically significant.

## Results

### IL-23R deficiency leads to temporal abnormalities in trabecular bone growth in mice

**Figure 1:**
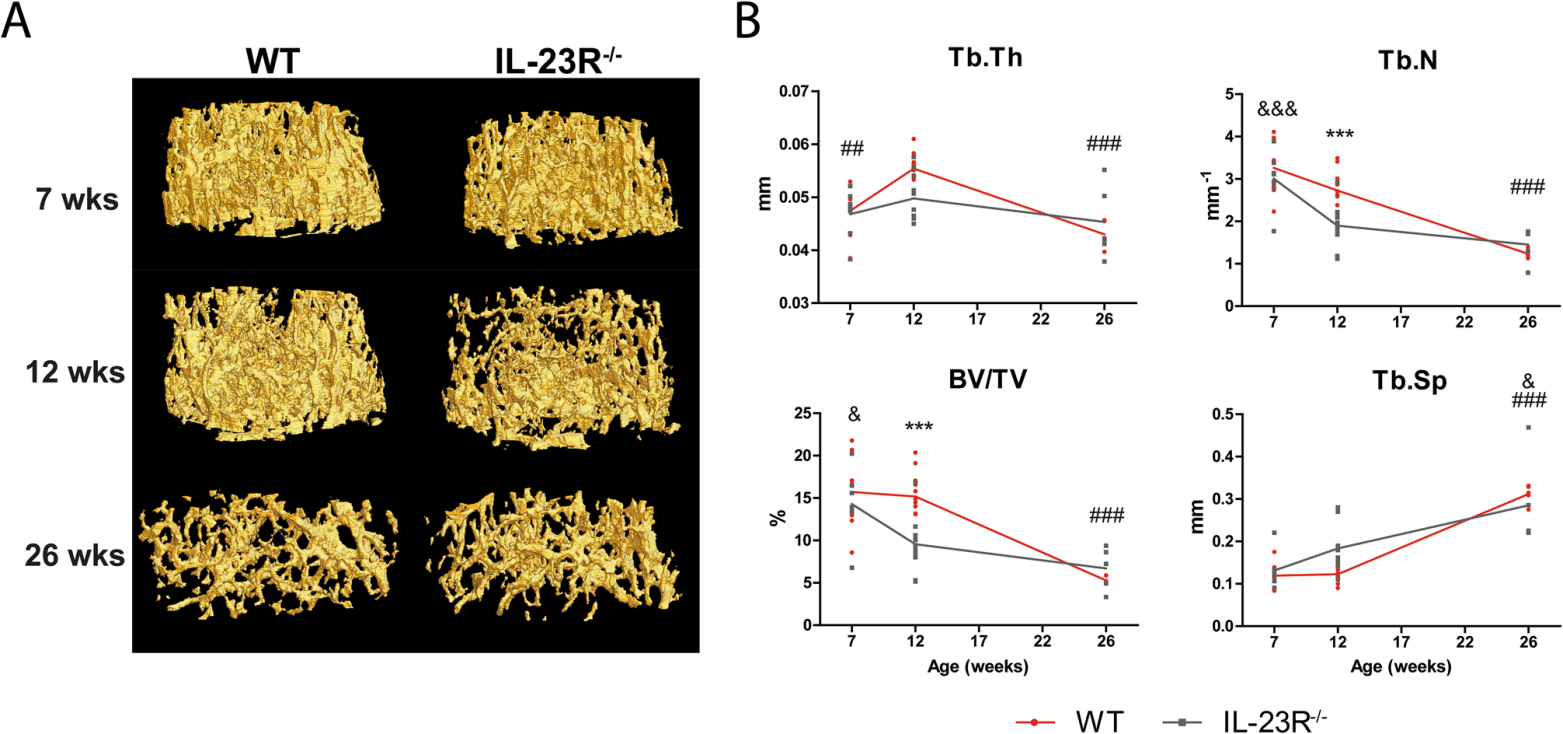
12-week-old IL-23R deficient mice have lower trabecular bone mass compared to WT. Femurs of 7-, 12- and 26-week-old WT and IL-23R^−/−^ mice were used for μCT analysis. (**A**) Representative images of µCT analysis of trabecular bone. (**B**) Trabecular bone mass parameters. *Tb.Th* trabecular thickness, *Tb.N* trabecular number, *BV/TV* bone volume fraction, *Tb.Sp* trabecular separation. Pooled data of n = 7 (7 weeks; two independent experiments), n = 11 (12 weeks; three independent experiments) and n = 5 (26 weeks; one experiment) mice per group. Data are depicted as mean ± SEM. *Significant difference between WT and IL-23R^−/−^ mice at indicated age, ^#^significantly different from 12 weeks within WT group, ^&^significantly different from 12 weeks within IL-23R^−/−^ group. ^&^ *p* < 0.05, ^##^*p* < 0.01, ^###^, *** or ^&&&^*p* < 0.001.

**Figure 2:**
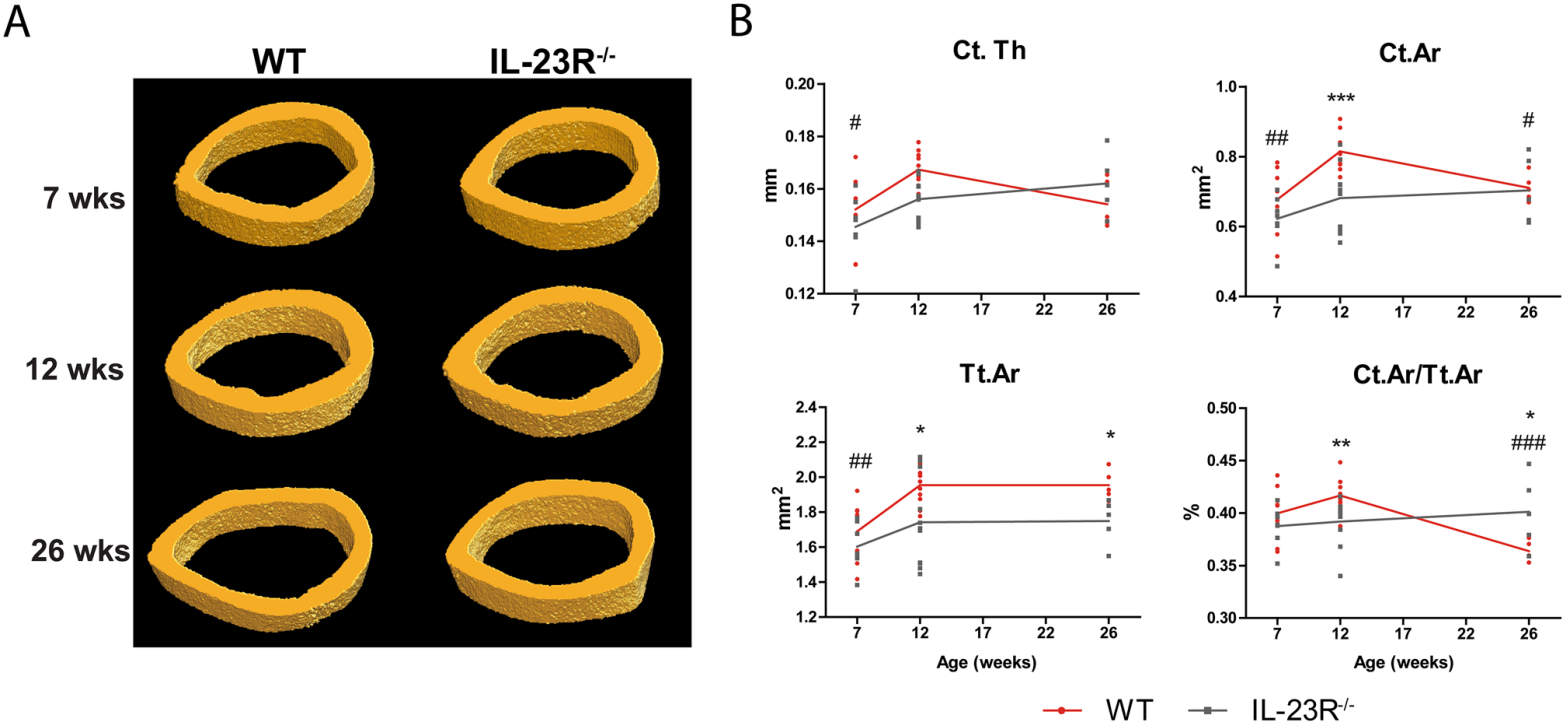
12-week-old IL-23R deficient mice have lower cortical bone mass compared to WT. Femurs of 7-, 12- and 26-week-old WT and IL-23R^−/−^ mice were used for μCT analysis (**A**) Representative images of µCT analysis of cortical bone. (**B**) Cortical bone mass parameters. *Ct.Th* average cortical thickness, *Ct.Ar* cortical bone area, *Tt.Ar* total cross-sectional area inside the periosteal envelope, *Ct.Ar/Tt.Ar* cortical area fraction. Pooled data of n = 7 (7 weeks; two independent experiments), n = 11 (12 weeks; three independent experiments) and n = 5 (26 weeks; one experiment) mice per group. Data are depicted as mean ± SEM. *Significant difference between WT and IL-23R^−/−^ mice at indicated age, ^#^significantly different from 12 weeks within WT group. * or ^#^*p* < 0.05, ^**^ or ^##^*p* < 0.01, ^###^ or ***p < 0.001.

Previously, Quinn et al*.* demonstrated that IL-23p19^−/−^ mice have reduced trabecular bone mineral density^17^. To investigate if IL-23R^−/−^ mice present similar phenotype, we subjected the femurs of 7-, 12- and 26-week-old male mice to a μCT analysis (Figs. 1, 2, Fig. S1). Trabecular bone parameters such as trabecular thickness (Tb.Th), number (Tb.N), bone volume fraction (BV/TV), separation (Tb.Sp) and structure model index (SMI) were similar between 7-week-old WT and IL-23R^−/−^ mice (Fig. 1, Fig. S1A; Table S1). However, differences in these parameters were most notable at 12 weeks of age between both groups. IL-23R^−/−^ BV/TV, Tb.Th, Tb.N and SMI were lower compared to WT, while Tb.Sp was higher (Fig. 1, Fig. S1A; Table S1). This difference can be explained by the stronger decrease in BV/TV (WT 3.5%; KO 33%) and Tb.N (WT 16%; KO 37%) of IL-23R^−/−^ mice between the age of 7 and 12 weeks compared to WT mice (Fig. 1B). Furthermore, Tb.Th increased significantly (14%; P < 0.01) in WT mice between 7 and 12 weeks, but this difference was not-significant (6%) in IL-23R deficient mice.

Surprisingly, trabecular bone mass was similar to WT in 26-week-old IL-23R^−/−^ mice. In WT mice, there was a significant decrease of 65% in BV/TV (P < 0.001), 54% in Tb.N (P < 0.001) and 22% in Tb.Th (P < 0.001) between 12 and 26 weeks, while in IL-23R^−/−^ mice there was a smaller decrease of 29% in BV/TV, 23% in Tb.N and 9% in Tb.Th. Above data demonstrate that IL-23R deficiency leads to temporal changes in trabecular bone mass parameters.

### IL-23R deficiency leads to long-term abnormalities in cortical bone growth in mice

Cortical bone mass parameters such as cortical thickness (Ct.Th), total cross-sectional area inside the periosteal envelope (Tt.Ar), cortical bone area (Ct.Ar), cortical area fraction (Ct.Ar/Tt.Ar), periosteal perimeter (Ps.Pm), endocortical perimeter (Ec.Pm) and medullary area (Ma.Ar) were not significantly different between femurs of WT and IL-23R^−/−^ mice at 7 weeks (Fig. 2, Fig. S1B; Table S1). At 12 weeks of age, Ct.Ar, Tt.Ar, Ct.Ar/Tt.Ar, Ps.Pm and Ec.Pm were significantly lower in IL-23R^−/−^ mice compared to WT. Between 7 and 12 weeks, Ct.Th (WT 9%; KO 6%), Ct.Ar (WT 17%; KO 9%), Tt.Ar (WT 14%; KO 8%), Ps.Pm (WT 7%; KO 4%), Ec.Pm (WT 8%; KO 4%), Ma.Ar (WT 11%; KO 6%) increased significantly in WT mice, however this increase was relatively small and not significant in IL-23R^−/−^ mice. In contrast, between the ages of 12 and 26 weeks, there was a significant decrease in Ct.Ar (13%; P < 0.05) and Ct.Ar/Tt.Ar (12%; P < 0.001) of WT femurs, while there was a slight increase in these perimeters in IL-23R^−/−^ mice (3% and 2% respectively). This resulted in similar Ct.Ar between both groups at 26 weeks and higher Ct.Ar/Tt.Ar in IL-23R^−/−^ mice. Notably, Tt.Ar (P < 0.05), Ps.Pm (P < 0.05), Ec.Pm (P < 0.01) and Ma.Ar (P < 0.01) were all significantly smaller in 26-week-old IL-23R^−/−^ mice compared to WT. These data demonstrate that IL-23R deficiency leads to long-term abnormalities of cortical bone growth.

### IL-23R deficient mice have shorter femurs at 12 weeks

**Figure 3:**
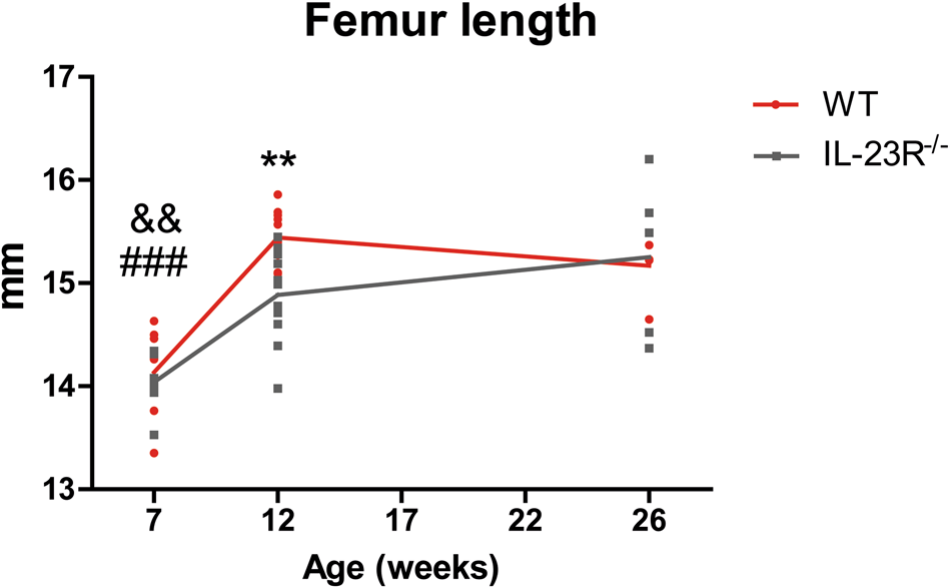
12-week-old IL-23R deficient mice have shorter femurs compared to WT. Femur length of 7-, 12- and 26-week-old WT and IL-23R^−/−^ mice was determined using μCT. Pooled data of n = 7 (7 weeks; two independent experiments), n = 11 (12 weeks; three independent experiments) and n = 5 (26 weeks; one experiment) mice per group. Data are depicted as mean ± SEM. *Significant difference between WT and IL-23R^−/−^ mice at indicated age, ^#^significantly different from 12 weeks within WT group, ^&^significantly different from 12 weeks within IL-23R^−/−^ group. ** or ^&&^*p* < 0.01, ^###^*p* < 0.001.

Femur length was similar between both groups at 7 weeks. In line with the lower bone mass at 12 weeks, IL-23R^−/−^ femurs were significantly shorter than those of WT mice (WT 15.4 ± 0.2 µm; KO 14.9 ± 0.4 µm; P < 0.01; Fig. 3). Interestingly, femur length in 26-week-old IL-23R^−/−^ mice was similar to WT mice. This suggests that IL-23R deficiency leads to temporal abnormalities in longitudinal bone growth.

### Femurs of 12-week-old IL-23R deficient mice are more brittle than WT

**Figure 4:**
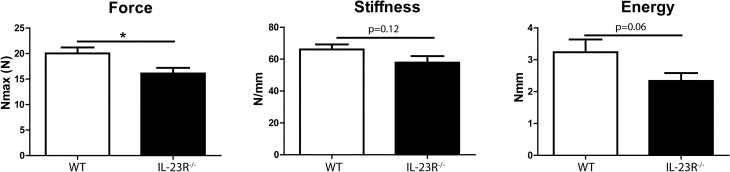
IL-23R^−/−^ mice have more brittle bones compared to WT at 12 weeks. Femurs of 12-week-old mice were subjected to three-point bending test for assessing bone strength. Pooled data of three independent experiments with n = 10–11 mice per group. Data are depicted as mean ± SEM. *p < 0.05*.*

The μCT data demonstrated that 12-week-old IL-23R^−/−^ mice have lower bone mass. To investigate if the mechanical properties of IL-23R^−/−^ femurs are also affected, we subjected the femurs to a three-point bending test. The total force required to fracture the bones was significantly lower in IL-23R^−/−^ femurs (WT 20 ± 3.7 N; KO 16.1 ± 3.7 N; P < 0.05; Fig. 4). In agreement, there was a clear trend towards lower stiffness and energy required to fracture (work-to-failure) in IL-23R^−/−^ femurs. Altogether, these data suggest that IL-23R deficiency leads to more fragile bones.

### Differentiation and function of IL-23R^−/−^ osteoclasts is unaltered at 7 and 12 weeks

Since bone mass was lower in 12-week-old IL-23R^−/−^ mice, we investigated if this was potentially induced by increased osteoclast development. To study if there were abnormalities in the osteoclast precursors of 12-week-old IL-23R^−/−^ mice, we analyzed their bone marrow by flow cytometry (Fig. S2). The percentage of early blasts and myeloid blasts did not differ between both groups (Fig. S2B). However, the fraction of monocytes was reduced in IL-23R^−/−^ BM.

**Figure 5:**
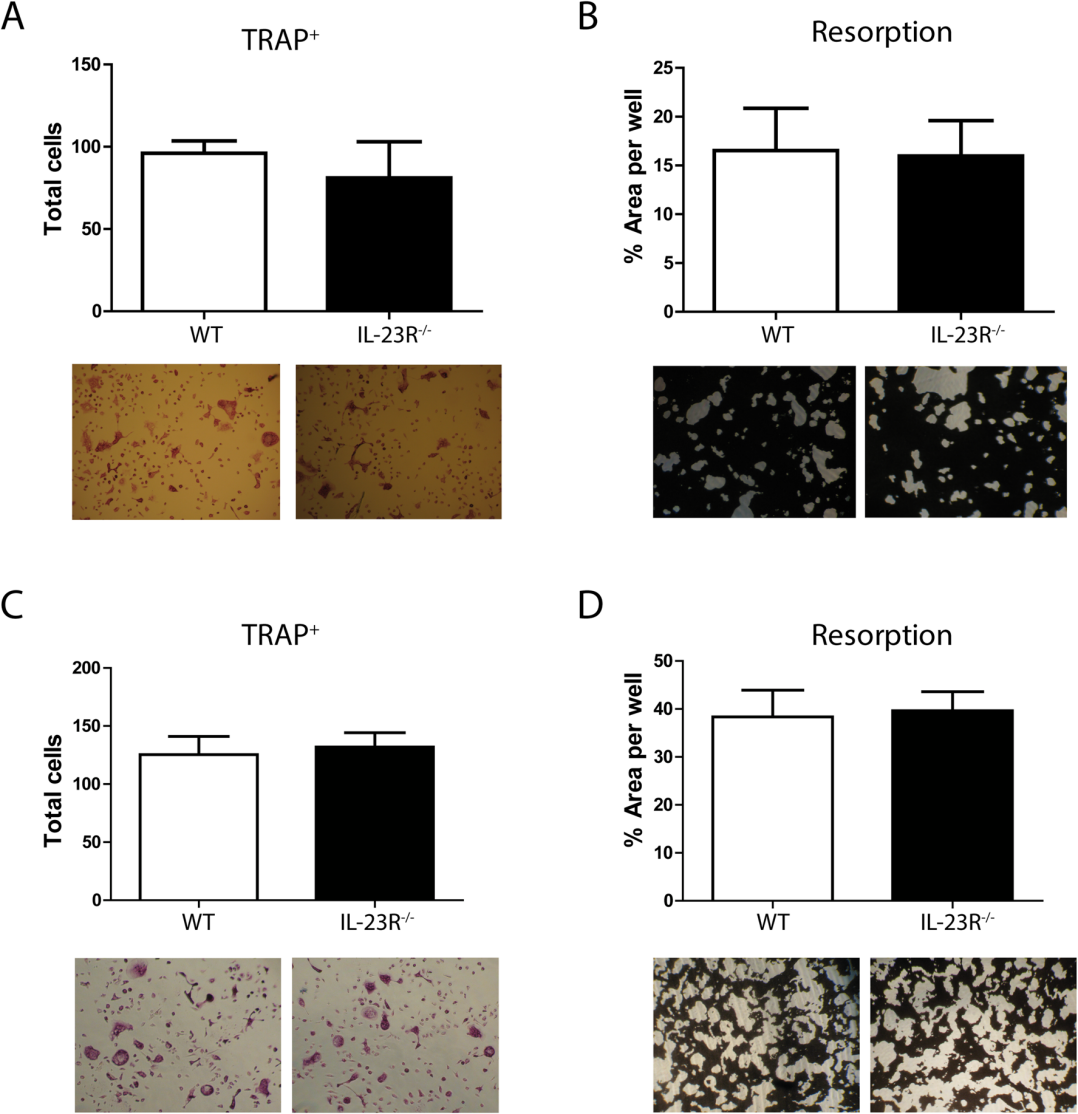
In vitro osteoclast differentiation and function of 7- and 12-week-old mice is unaltered in the absence of IL-23R. BM cells of 7- or 12-week-old mice were stimulated to differentiate towards osteoclasts with M-CSF and RANKL. Cells were assessed for osteoclasts at day 5 of culture via TRAP staining. Osteoclast function was assessed at day 7 (12-week-old) or 9 (7-week-old) of culture with resorption assay. (**A**) Trap staining at 7 weeks. (**B**) Bone resorption at 7 weeks. Pooled data of two independent experiments with n = 7 mice per group. (**C**) Trap staining at 12 weeks. (**D**) Bone resorption at 12 weeks. Representative of three independent experiments with n = 3–5 mice per group per experiment.

Next, we determined the ability of the BM cells to differentiate towards osteoclasts in vitro upon stimulation with RANKL and M-CSF at 7 and 12 weeks. We detected equal numbers of TRAP^+^ cells, with similar sizes, in both WT and IL-23R^−/−^ cultures (Fig. 5A,C).

In addition, the bone resorptive capacity of IL-23R^−/−^ osteoclasts was similar to WT (Fig. 5B,D). In summary, these data demonstrate that IL-23R deficiency does not affect osteoclast function and differentiation in 7- and 12-week-old mice.

### Bone formation marker PINP is lower in the serum of 12-week-old IL-23R^−/−^ mice compared to WT

**Figure 6:**
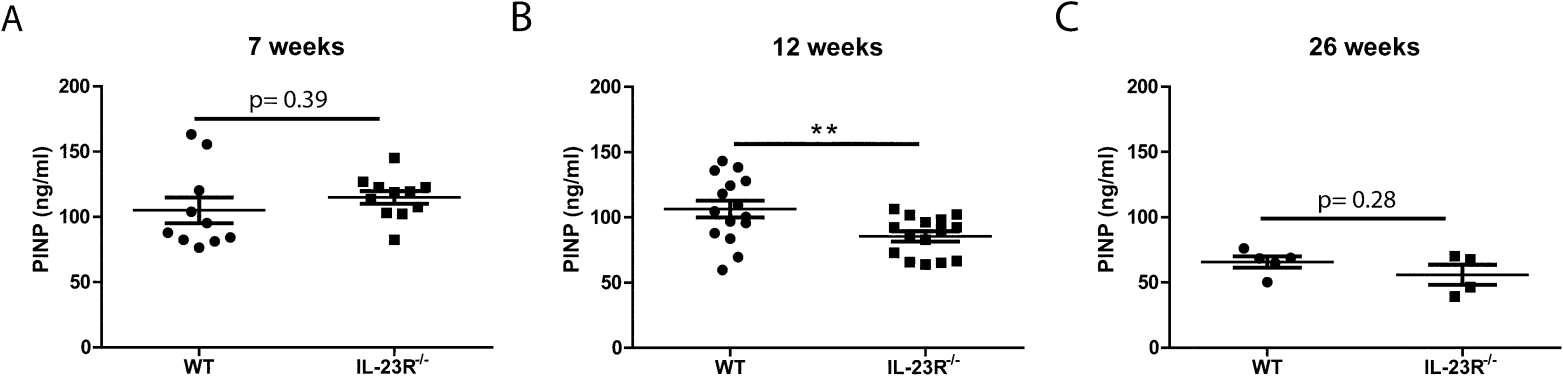
Serum PINP is significantly lower in 12-week-old IL-23R^−/−^ mice compared to WT. Serum PINP was measured in (**A**) 7-week-old, (**B**) 12-week-old or (**C**) 26-week-old WT and IL-23R^−/−^ mice. Pooled data of n = 10–11 7-week-old mice (three independent experiments), n = 15 12-week-old mice (four independent experiments) and n = 4–5 26-week-old mice (one experiment). Data are depicted as mean ± SEM. **p < 0.01.

Next, we assessed serum levels of the bone turnover marker PINP in our mice. At 7 weeks, there was no difference between both groups (Fig. 6A). However, at 12 weeks, IL-23R^−/−^ mice had significantly lower PINP levels compared to WT (Fig. 6B). At 26 weeks, PINP levels were similar between the two groups (Fig. 6C). To determine if IL-23 can act directly on osteoblasts, we cultured BM cells towards osteoblasts and analyzed gene expression of the IL-23R subunits in these cells at day 10 of culture. Although *Il12rβ1*was expressed similarly in osteoblasts of both genotypes, *Il23r* could not be detected (Fig. S3). Combined, these data suggest that IL-23R signaling affects osteoblast activity indirectly.

### Serum leptin is similar between 7- and 12-week-old WT and IL-23R^−/−^ mice

Our data suggest that the effects of IL-23 on osteoblasts are indirect, therefore, other factors are likely involved. To determine whether an altered gonadal function might be involved, we measured serum levels of testosterone and Anti-Müllerian hormone, but did not detect differences between both groups (data not shown).

**Figure 7:**
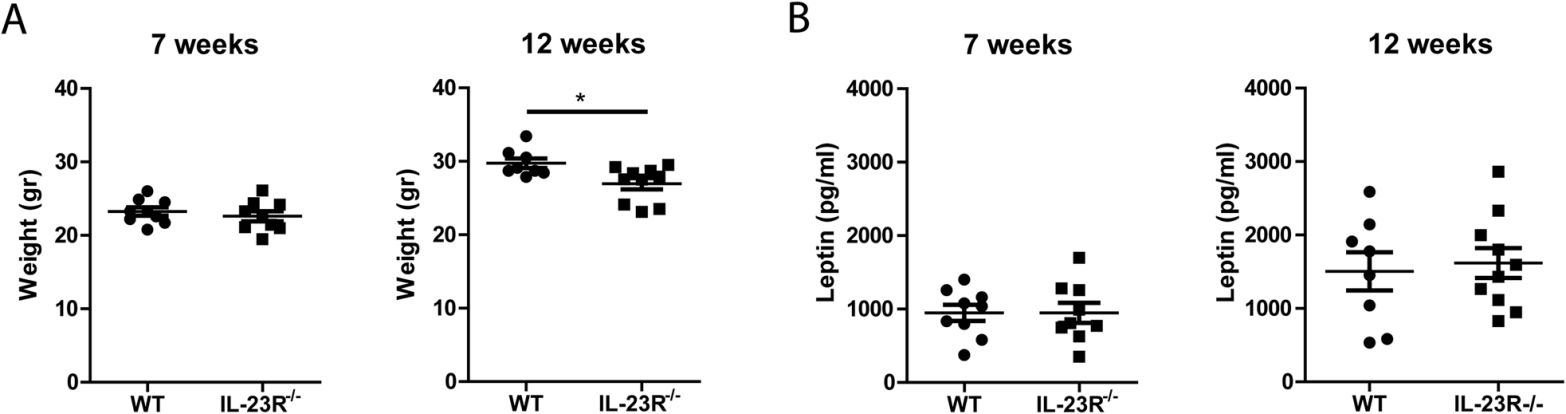
Serum leptin is similar to WT in 7- and 12-week-old IL-23R deficient mice. Seven- and 12-week-old WT and IL-23R^−/−^ mice were weighed and serum was used for leptin measurement. (**A**) Weight of mice in grams. (**B**) Leptin measured in serum by ELISA. Pooled data of n = 9 mice (7 weeks) per group from two independent experiments, and n = 8–10 mice per group (12 weeks) from three independent experiments. Data are depicted as mean ± SEM. *p < 0.05.

Next, we investigated whether absence of IL-23R affected the metabolic system by assessing the body weight of the mice at 7, 12 and 26 weeks. Notably, 12-week-old, but not 7- and 26-week-old, IL-23R^−/−^ mice had significantly lower body weight than WT mice (Fig. 7A, Fig. S4). This difference in weight prompted us to measure leptin levels in the mice. We detected similar serum levels of leptin in 7- and 12-week-old IL-23R^−/−^ mice compared to WT (Fig. 7B). Our data suggest that testosterone, AMH and leptin are not involved in IL-23R signaling-mediated regulation of osteoblasts.

## Discussion

In this study, we demonstrate that 12-week-old IL-23R deficient mice have reduced bone mass, more fragile bones and shorter femurs compared to WT. While trabecular bone mass is restored at 26 weeks, the effects on cortical bone parameters seem of long-term. No changes were found in osteoclasts of 7- or 12-week-old IL-23R^−/−^ mice. However, serum PINP levels were significantly lower in 12-week-old IL-23R deficient mice. The effects of IL-23R signaling on osteoblasts are most likely indirect, since the *il23r* subunit was not expressed in these cells. Apparently, mice lacking IL-23R undergo systemically driven temporal changes in osteoblast function, for which the exact cause yet has to be identified. These changes result into temporal and long-term alterations of the bone.

Our finding that 7-week-old IL-23R deficient mice have similar bone mass as WT mice, is in agreement with the study of Adamopoulos et al., who did not find any bone abnormalities in 8-week-old IL-23p19^−/−^ mice^14^. Furthermore, no defects in bone mass were found in 4-week-old IL-23p19^−/−^ mice^17^, suggesting that bone abnormalities due to disruption of the IL-23/IL-23R signaling develop at later ages. Indeed, 12-week-old IL-23R deficient mice had significantly less bone mass compared to WT, which is in line with the previously reported trabecular bone phenotype in IL-23p19^−/−^ mice of similar age^17^. In contrast to our study and that of Quinn et al*.*, Sato et al. did not find any bone abnormalities in 12-week-old IL-23p19^−/−^ mice^18^.

Earlier studies have demonstrated that cortical bone plays a major role in determining the mechanical properties of the bone and the risk of fracture, since most of fragility fractures occurs at non-vertebral sites where bone is composed mainly by cortical tissue^34^. To our knowledge, we demonstrate here for the first time that cortical bone mass is affected in the absence of IL-23R at 12 weeks, and that the bones of IL-23R deficient mice are more fragile than WT.

We found that trabecular bone mass and femur length of 26-week-old IL-23R^−/−^ mice were similar to WT. This seemed to be induced due to reduced bone loss in IL-23R^−/−^ mice between the age of 12 and 26 weeks. Supporting this, a recent study demonstrated higher bone mass in 12-months-old IL-12p40^−/−^ mice, which lack both IL-12 and IL-23, suggesting that lack of IL-23 has a protective role in age-related bone loss^19^. In contrast, Quinn et al. reported reduced BV/TV, Tb.N and Tb.Th in 26-week-old IL-23p19^−/−^ mice^17^, while Adamopoulos et al. reported no significant differences in BV/TV, but higher Tb.N in 26-week-old IL-23p19^−/−^ mice^14^. These differences could be due to the use of equipment with different sensitivities for bone mass determination, differences in the genetic background of the mice and/or differences in used control mice (littermate vs non-littermate). Additionally, due to reduction in trabecular bone mass at older ages, detection of small differences in bone mass could be more challenging. Nonetheless, all these studies demonstrate that age is an important factor in the IL-23-dependent effects on the bone.

In contrast to trabecular bone, the effects of IL-23R deficiency on cortical bone including radial bone growth seemed to be of long-term. Considering the different effects of IL-23R deficiency on longitudinal (temporal) versus radial bone growth (long-term), it would be interesting to follow these mice for longer periods to obtain a better understanding of their phenotype at older ages.

In contrast to osteoclasts, our data suggest the involvement of osteoblasts in the IL-23R-dependent regulation of bone mass. Notably, PINP levels were not different at 7 and 26 weeks, but were significantly lower in 12-week-old IL-23R^−/−^ mice compared to WT. PINP is a bone formation marker that is used by a large number of studies as a good clinical marker of bone metabolism related diseases^35^. Serum PINP levels correlated well with the bone phenotype of the mice over time, suggesting that the observed lower bone mass is due to defects in bone formation. The notion that osteoblast-related factors are involved in the effects of IL-23 on the bone, are supported by a recent study which reported decrease in ALP activity in mice lacking IL-12p40^19^.

Interestingly, the effects of the IL-23/IL-23R signaling pathway on osteoblasts seem to be indirect, since we did not detect expression of *Il23r* subunit on osteoblasts. This is in line with previous studies which have reported lack of IL-23R on osteoblasts and no direct effect of IL-23 stimulation on these cells^17,24^. To study which factors were involved in the indirect effects of IL-23R signaling on osteoblasts, we assessed body weight, serum levels of AMH, testosterone and leptin. The lower bone mass phenotype and body weight of 12-week-old IL-23R^−/−^ mice was not due to systemic changes in AMH, testosterone or leptin levels. Future studies should reveal whether IL-23R signaling has a role on other aspects of osteoblast function, and what is causing the effects of IL-23R deficiency on the bone formation to occur at 12 weeks, but not at earlier or later ages. Also, it should be investigated whether the lower body weight at 12 weeks is due to lower bone mass or whether other factors are involved.

The bone phenotype observed at 12 weeks of age in IL-23R^−/−^ mice may resemble an osteoporotic phenotype. Our data support the study of Azizieh et al*.*, who found lower IL-23 levels produced by peripheral blood mononuclear cells of osteoporotic women compared to women with normal bone mineral density^36^. This suggests that low IL-23 levels could serve as a marker for bone loss in these patients. On the other hand, in PsA patients, treatment with anti-IL-23 biologicals has been demonstrated effective in reduction of disease activity and consequently halting inflammation-induced bone erosions^37^. However, the effects of long-term anti-IL-23 biological treatment on (systemic) bone mass and excessive local bone formation has not been clearly established.

It should be noted that our study has a few limitations. We studied the effects of IL-23R deficiency on the bone until 26 weeks of age. Despite having studied these mice at 3 different ages and revealing both temporary and long-term effects of IL-23R deficiency, it would be interesting to study the long-term effects on bone mass and strength at later ages after 26 weeks.

Moreover, we used in vitro differentiated cells and since cultured cells do not necessarily reflect the in vivo situation, further in vivo studies should be performed to understand the effects of IL-23R deficiency on bone mass.

In conclusion, we demonstrate here that IL-23R deficient mice have a temporary defect in their bone formation, which results in temporal effects on trabecular bone and long-term effects on cortical bone. Our study points towards possible implications for patients with long term treatment with anti-IL-23 biologicals who may suffer from loss of bone mass due to prolonged decrease in IL-23 levels.

## Supplementary Information

Supplementary Information 1.

## Abbreviations

IL-23R: Interleukin-23 receptor
RANK: Receptor activator of nuclear factor-κB
BM: Bone marrow
TRAP: Tartrate-resistant acid phosphatase
RA: Rheumatoid arthritis
PsA: Psoriatic arthritis
PINP: Procollagen I N-terminal propeptide

## References

[1] Chen X (2018). Osteoblast–osteoclast interactions. Connect Tissue Res..

[2] Ponzetti M, Rucci N (2019). Updates on osteoimmunology: What's new on the cross-talk between bone and immune system. Front. Endocrinol..

[3] Raterman HG, Lems WF (2019). Pharmacological management of osteoporosis in rheumatoid arthritis patients: A review of the literature and practical guide. Drugs Aging.

[4] Corrado A, Neve A, Maruotti N, Cantatore FP (2013). Bone effects of biologic drugs in rheumatoid arthritis. Clin. Dev. Immunol..

[5] Gravallese EM, Schett G (2018). Effects of the IL-23–IL-17 pathway on bone in spondyloarthritis. Nat. Rev. Rheumatol..

[6] Chisălău BA (2020). New insights into IL-17/IL-23 signaling in ankylosing spondylitis (Review). Exp. Ther. Med..

[7] Gravallese EM, Schett G (2018). Effects of the IL-23-IL-17 pathway on bone in spondyloarthritis. Nat. Rev. Rheumatol..

[8] Alsheikh MM, El-Shafey AM, Gawish HH, El-Desoky ET (2019). Serum interleukin-23 level in rheumatoid arthritis patients: Relation to disease activity and severity. Egypt Rheumatol..

[9] Oppmann B (2000). Novel p19 protein engages IL-12p40 to form a cytokine, IL-23, with biological activities similar as well as distinct from IL-12. Immunity.

[10] Khader SA, Thirunavukkarasu S (2019). The tale of IL-12 and IL-23: A paradigm shift. J. Immunol..

[11] Parham C (2002). A receptor for the heterodimeric cytokine IL-23 is composed of IL-12R beta 1 and a novel cytokine receptor subunit, IL-23R. J. Immunol..

[12] Bloch Y (2018). Structural activation of pro-inflammatory human cytokine IL-23 by cognate IL-23 receptor enables recruitment of the shared receptor IL-12Rbeta1. Immunity.

[13] Razawy W, van Driel M, Lubberts E (2018). The role of IL-23 receptor signaling in inflammation-mediated erosive autoimmune arthritis and bone remodeling. Eur. J. Immunol..

[14] Adamopoulos IE (2011). IL-23 is critical for induction of arthritis, osteoclast formation, and maintenance of bone mass. J. Immunol..

[15] Chen L (2020). Skin expression of IL-23 drives the development of psoriasis and psoriatic arthritis in mice. Sci. Rep..

[16] Sherlock JP (2012). IL-23 induces spondyloarthropathy by acting on ROR-gammat+ CD3+CD4-CD8^−^ entheseal resident T cells. Nat. Med..

[17] Quinn JM (2008). IL-23 inhibits osteoclastogenesis indirectly through lymphocytes and is required for the maintenance of bone mass in mice. J. Immunol..

[18] Sato K (2006). Th17 functions as an osteoclastogenic helper T cell subset that links T cell activation and bone destruction. J. Exp. Med..

[19] Xu J (2020). IL-23, but not IL-12, plays a critical role in inflammation-mediated bone disorders. Theranostics.

[20] Chimenti MS (2020). Tackling the autoimmune side in spondyloarthritis: A systematic review. Autoimmun. Rev..

[21] Chen L, Wei XQ, Evans B, Jiang W, Aeschlimann D (2008). IL-23 promotes osteoclast formation by up-regulation of receptor activator of NF-kappaB (RANK) expression in myeloid precursor cells. Eur. J. Immunol..

[22] de Vries TJ, Schoenmaker T, Hooibrink B, Leenen PJM, Everts V (2009). Myeloid blasts are the mouse bone marrow cells prone to differentiate into osteoclasts. J. Leukocyte Biol..

[23] Kamiya S (2007). Effects of IL-23 and IL-27 on osteoblasts and osteoclasts: Inhibitory effects on osteoclast differentiation. J. Bone Miner. Metab..

[24] Zhang JR (2017). Different modulatory effects of IL-17, IL-22, and IL-23 on osteoblast differentiation. Mediators Inflamm..

[25] El-Zayadi AA (2017). Interleukin-22 drives the proliferation, migration and osteogenic differentiation of mesenchymal stem cells: A novel cytokine that could contribute to new bone formation in spondyloarthropathies. Rheumatology.

[26] Mohamad NV, Soelaiman IN, Chin KY (2016). A concise review of testosterone and bone health. Clin. Interv. Aging.

[27] Upadhyay J, Farr OM, Mantzoros CS (2015). The role of leptin in regulating bone metabolism. Metabolism.

[28] Goto K (2015). Leptin deficiency down-regulates IL-23 production in glomerular podocytes resulting in an attenuated immune response in nephrotoxic serum nephritis. Int. Immunol..

[29] Martins LMS (2018). Interleukin-23 promotes intestinal T helper type17 immunity and ameliorates obesity-associated metabolic syndrome in a murine high-fat diet model. Immunology.

[30] Awasthi A (2009). Cutting edge: IL-23 receptor GFP reporter mice reveal distinct populations of IL-17-producing cells. J. Immunol..

[31] Bouxsein ML (2010). Guidelines for assessment of bone microstructure in rodents using micro-computed tomography. J. Bone Miner. Res..

[32] van der Eerden BC (2013). TRPV4 deficiency causes sexual dimorphism in bone metabolism and osteoporotic fracture risk. Bone.

[33] Razawy W (2017). Experimental arthritis mouse models driven by adaptive and/or innate inflammation. Methods Mol. Biol..

[34] Iolascon G (2013). The contribution of cortical and trabecular tissues to bone strength: Insights from denosumab studies. Clin. Cases Miner. Bone Metab..

[35] Krege JH, Lane NE, Harris JM, Miller PD (2014). PINP as a biological response marker during teriparatide treatment for osteoporosis. Osteopor. Int..

[36] Azizieh F, Raghupathy R, Shehab D, Al-Jarallah K, Gupta R (2017). Cytokine profiles in osteoporosis suggest a proresorptive bias. Menopause.

[37] Kavanaugh A (2014). Ustekinumab, an anti-IL-12/23 p40 monoclonal antibody, inhibits radiographic progression in patients with active psoriatic arthritis: Results of an integrated analysis of radiographic data from the phase 3, multicentre, randomised, double-blind, placebo-controlled PSUMMIT-1 and PSUMMIT-2 trials. Ann. Rheum. Dis..

