# Supplementary material for: IL-23 receptor deficiency results in lower bone mass via indirect regulation of bone formation
Source: Sci Rep. 2021 May 13;11:10244. doi: 10.1038/s41598-021-89625-2 (PMC8119722; doi:10.1038/s41598-021-89625-2)
Supplement: Supplementary file 1 — Supplementary Information 1. [file 41598_2021_89625_MOESM1_ESM.pdf]

IL-23 receptor deficiency results in lower bone mass via indirect regulation of bone formation

Wida Razawy[1,2], Celso H. Alves[1,2#], Marijke Koedam[3], Patrick S. Asmawidjaja[1,2], Adriana M.C. Mus[1,2], Mohamed Oukka[4,5], Pieter J.M. Leenen[2], Jenny A. Visser[3], Bram C.J. van der Eerden[3], Erik Lubberts[1,2*]

Departments of [1]Rheumatology, [2]Immunology and [3]Internal Medicine, Erasmus MC, University Medical Center Rotterdam, Rotterdam, The Netherlands

[4]Dept. of Pediatrics, Seattle Children's Research Institute, Center for Immunity and Immunotherapies, Seattle, USA

[5]Dept. of Immunology, University of Washington, Seattle, USA

#Current address: [1]University of Coimbra, Center for Innovative Biomedicine and Biotechnology (CIBB), Coimbra, Portugal; [2]University of Coimbra, Coimbra Institute for Clinical and Biomedical Research (iCBR), Faculty of Medicine, Coimbra, Portugal; [3]Association for Innovation and Biomedical Research on Light and Image (AIBILI), Coimbra, Portugal; [4]Clinical Academic Center of Coimbra (CACC), Coimbra, Portugal

*Corresponding author:

Dr. Erik Lubberts, Erasmus MC, University Medical Center Rotterdam, Department of Rheumatology, Dr. Molewaterplein 40, 3015 GD, Rotterdam

Fax: +31-10-704-4593

e-mail: e.lubberts@erasmusmc.nl

Supplementary Information

Table S1. Trabecular and cortical bone parameters. Femurs of 7-, 12- and 26-week-old WT and IL-23R$^{-/-}$ mice were used for µCT analysis. Pooled data of n=7 (7 weeks; two independent experiments), n=11 (12 weeks; three independent experiments) and n=5 (26 weeks; one experiment) mice per group. Data are depicted as mean ± SD. *significantly different from WT at indicated age, $^{\#}$ significantly different from 12 weeks within WT group, $^{\&}$ significantly different from 12 weeks within IL-23R$^{-/-}$ group. *, $^{\#}$ *or* $^{\&}$*p<0.05; *** *or* $^{\#\#}$ *p< 0.01; ****,* $^{\#\#\#}$ *or* $^{\&\&\&}$*p<0.001.*

	7 weeks (mean ± SD)		12 weeks (mean ± SD)		26 weeks (mean ± SD)	
**Bone parameter**	**WT**	**IL-23R$^{-/-}$**	**WT**	**IL-23R$^{-/-}$**	**WT**	**IL-23R$^{-/-}$**
**Tb.Th (mm)**	0.047 ± 0.005; ##	0.046 ± 0.004	0.055 ± 0.003	0.049 ± 0.004	0.043 ± 0.002; ###	0.045 ± 0.006
**Tb.N (mm$^{-1)}$**	3.26 ± 0.63	3.00 ± 0.60; &&&	2.72 ± 0.48	1.90 ± 0.47; ***	1.23 ± 0.10; ###	1.45 ± 0.37
**BV/TV (%)**	15.72 ± 4.37	14.27 ± 3.78; &	15.18 ± 3.08	9.56 ± 3.03; ***	5.29 ± 0.31; ###	6.70 ± 2.27
**Tb.Sp (mm)**	0.12 ± 0.03	0.13 ± 0.04	0.12 ± 0.03	0.18 ± 0.05	0.31 ± 0.02; ###	0.29 ± 0.09; &
**SMI**	1.74 ± 0.25	1.91 ± 0.20	1.79 ± 0.17	2.14 ± 0.21; ***	2.47 ± 0.06; ###	2.39 ± 0.18
**Ct.Th (mm)**	0.15 ± 0.01; #	0.15 ± 0.01	0.17 ± 0.01	0.16 ± 0.01	0.15 ± 0.01	0.16 ± 0.01
**Ct.Ar (mm$^{2)}$**	0.68 ± 0.09; ##	0.62 ± 0.06	0.81 ± 0.05	0.68 ± 0.09; ***	0.71 ± 0.03; #	0.70 ± 0.09
**Tt.Ar (mm$^{2)}$**	1.69 ± 0.17; ##	1.60 ± 0.13	1.95 ± 0.10	1.74 ± 0.24; *	1.95 ± 0.07	1.74 ± 0.11; *
**Ct.Ar/Tt.Ar (%)**	0.40 ± 0.03;	0.39 ± 0.02	0.42 ± 0.01	0.39 ± 0.02; **	0.36 ± 0.01; ###	0.40 ± 0.03; *
**Ps.Pm (mm)**	5.02 ± 0.26; ##	4.89 ± 0.20	5.41 ± 0.14	5.11 ± 0.33; *	5.48 ± 0.10	5.17 ± 0.16; *
**Ec.Pm (mm)**	4.29 ± 0.20; #	4.13 ± 0.22	4.69 ± 0.31	4.31 ± 0.32; *	4.68 ± 0.13	4.26 ± 0.17; **
**Ma.Ar (mm$^{2)}$**	0.98 ± 0.10; #	0.95 ± 0.06	1.11 ± 0.07	1.01 ± 0.16	1.17 ± 0.05	0.98 ± 0.08 **

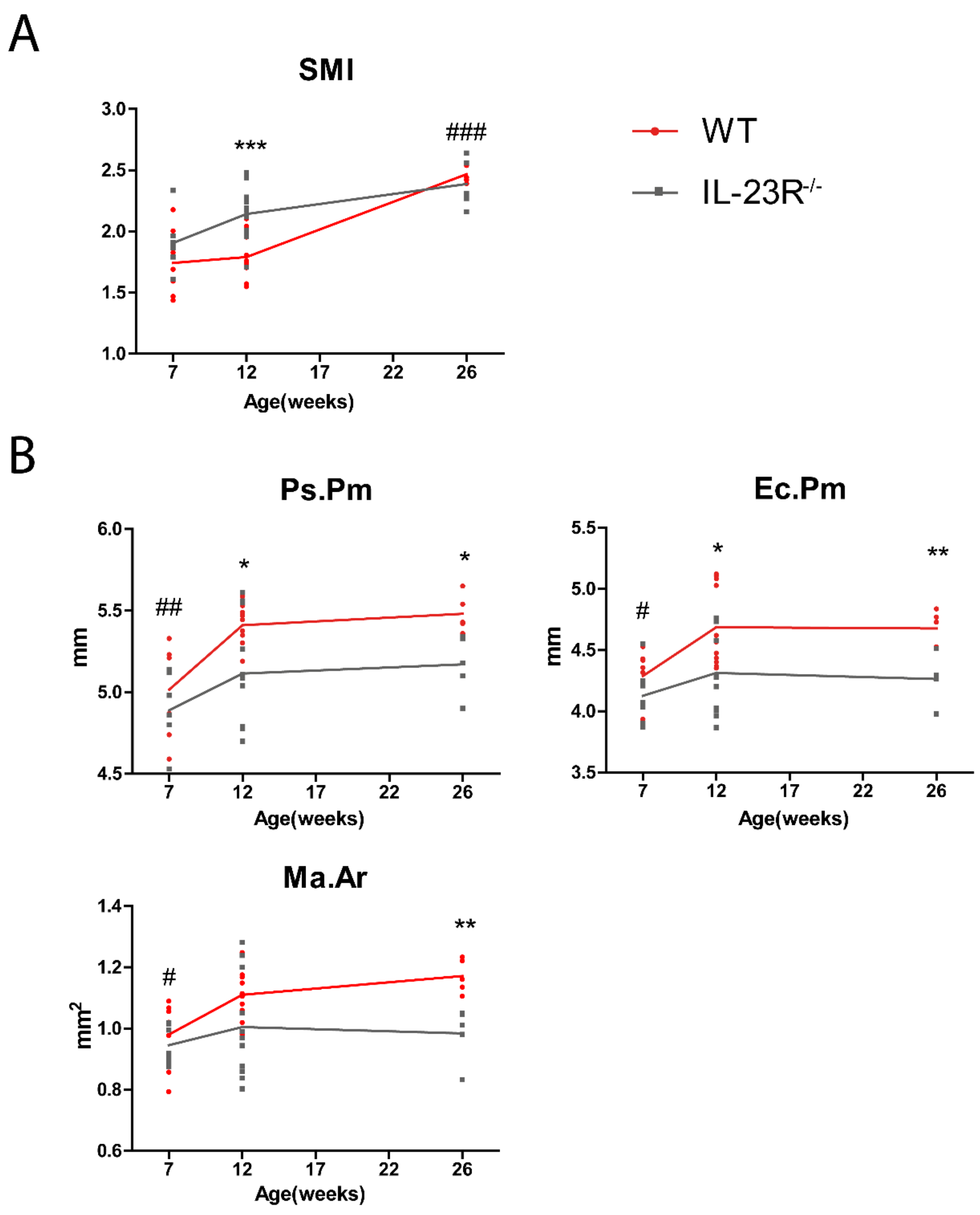

Figure S1. 12-week-old IL-23R deficient mice have lower bone mass compared to WT. Femurs of 7-, 12- and 26-week-old WT and IL-23R$^{-/-}$ mice were used for µCT analysis. **(A)** Trabecular bone mass parameter SMI: structure model index. **(B)** Cortical bone mass parameters. Ps.Pm: periosteal perimeter, Ec.Pm: endocortical perimeter, Ma.Ar: medullary area. Pooled data of n=7 mice per group (7 weeks; two independent experiments), n=11 mice per group (12 weeks; three independent experiments) and n=5 mice per group (26 weeks; one experiment). Data are depicted as mean ± SEM. *significant difference between WT and IL-23R$^{-/-}$ mice at indicated age, $^{\#}$significantly different from 12 weeks within WT group. * *or* $^{\#}p<0.05$, ** or $^{\#\#}p< 0.01$, ****or* $^{\#\#\#}p<0.001$.

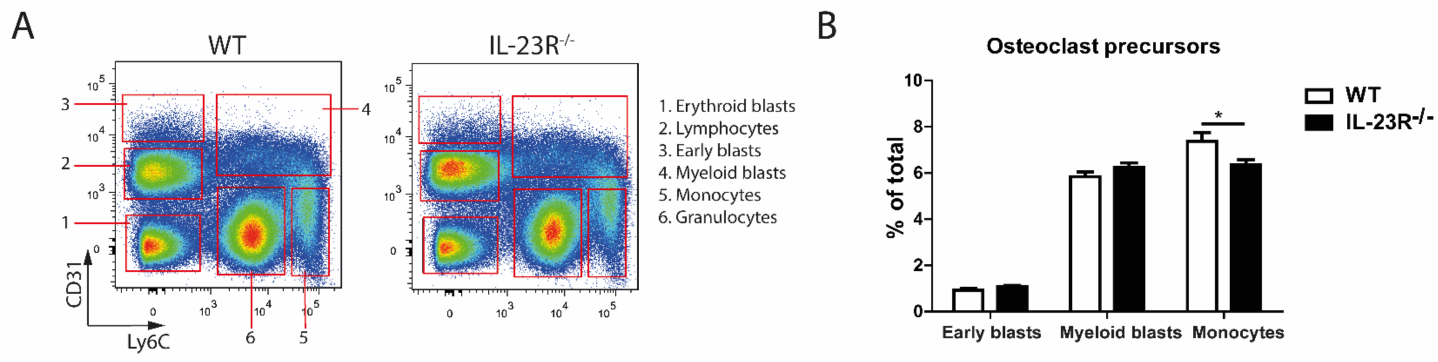

Figure S2. Osteoclast precursors of 12-week-old IL-23R$^{-/-}$ mice are similar to WT. BM cells of 12-week-old mice were used for assessment of osteoclast precursors. **(A)** Flow cytometry analysis of different BM populations based on CD31 and Ly6C expression after pre-gating on total live cells. **(B)** Quantification of (A). Data of n=5 mice per group are shown as mean ± SEM. **p<0.05.*

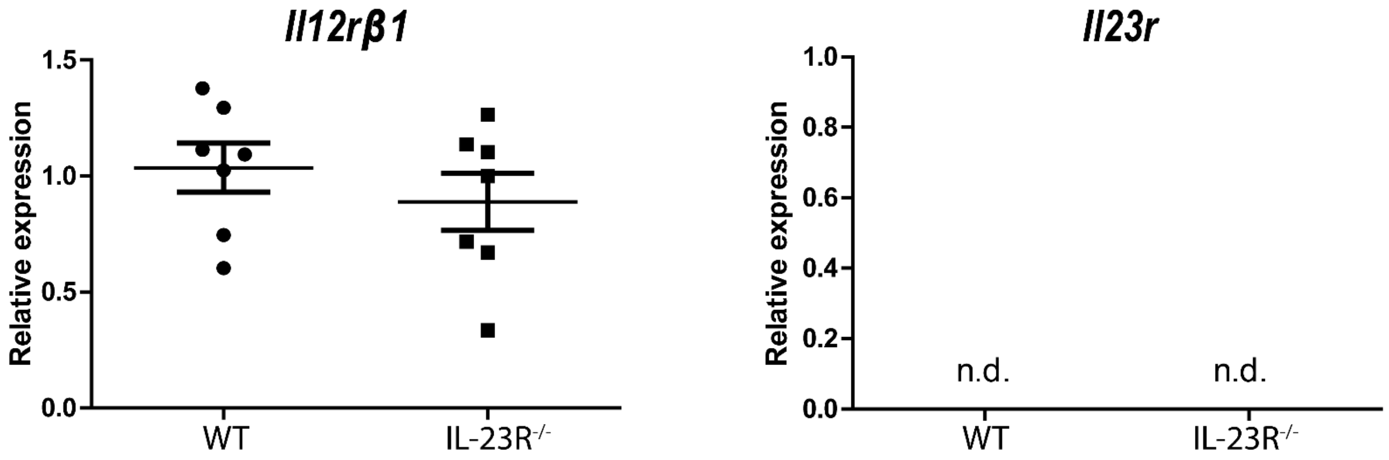

Figure S3. *Il23r* is not expressed in osteoblasts. Relative gene expression of *Il23r* and *Il12rβ1* measured in osteoblasts of 7-week-old mice at day 10 of culture determined by RT-PCR. Gene expression was normalized against GAPDH. Pooled data of two independent experiments with n=7 mice per group are depicted as mean ± SEM.

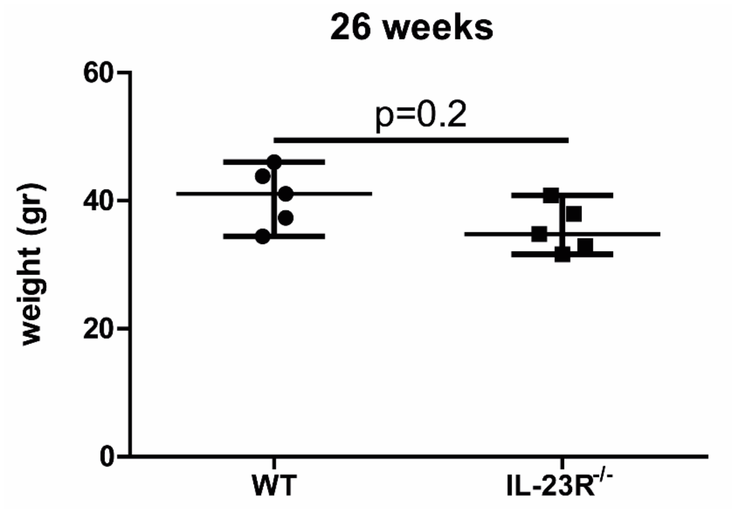

Figure S4. Body weight is similar between 26-week-old IL-23R$^{-/-}$ and WT mice. Data of n=5 WT and IL-23R$^{-/-}$ mice are depicted as mean ± SEM.
